# Supplementary figures and images for: The limitations of automatically generated curricula for continual learning
Source: PLoS One. 2024 Apr 16;19(4):e0290706. doi: 10.1371/journal.pone.0290706 (PMC11020929; doi:10.1371/journal.pone.0290706)

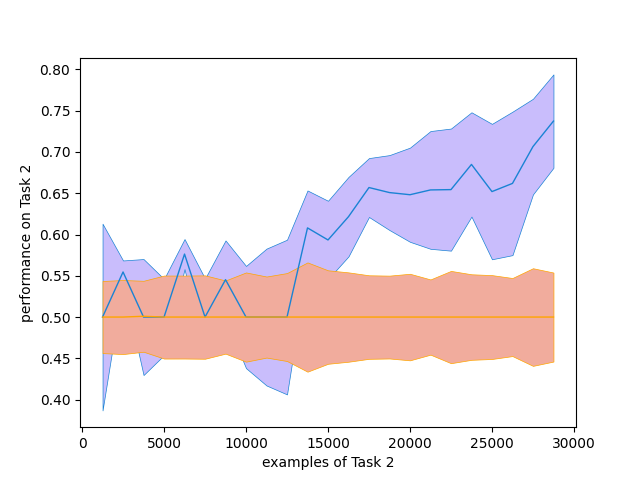

Supplement: S1 Fig — Doubling the amount of epochs made the task learnable, even if performance wasn’t as good as with the addition of pre-training on Task 1. With the original amount of epochs performance on Task 2 without pre-training averages to random with respect to some noise. In some learning cases there was a pattern of cycling between 40% and 60% accuracy (we attribute this effect to classes where the same digit repeats twice, the result then depends whether the network associated the visual features of this digit with the odd or even class). Again, in multiple runs this averages to 50% accuracy. (PNG) [file pone.0290706.s001.png]

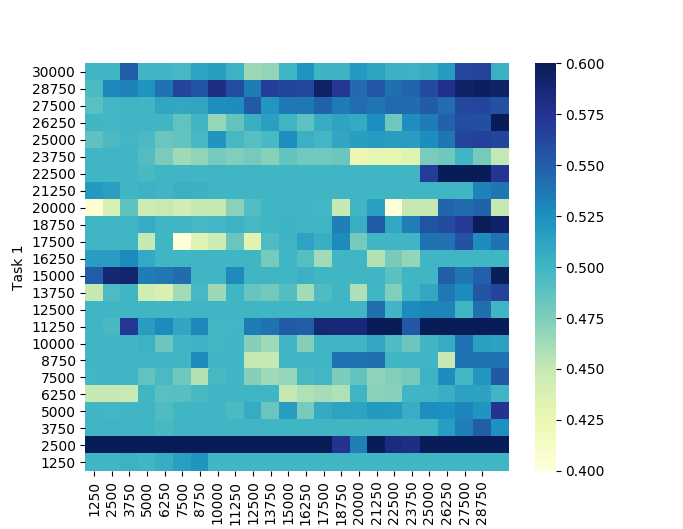

Supplement: S2 Fig — Class-incremental learning during pre-training led to a catastrophic forgetting scenario where the network never learned single digits representation enough to facilitate learning of Task 2. Due to the limitations of architecture imposed by the original study goal, ours isn’t a suitable model to deal with true non-stationarity and catastrophic forgetting. Investigating the interplay between curriculum and active learning will be the direction of our future work. (PNG) [file pone.0290706.s002.png]
