# Supplementary material for: The limitations of automatically generated curricula for continual learning
Source: PLoS One. 2024 Apr 16;19(4):e0290706. doi: 10.1371/journal.pone.0290706 (PMC11020929; doi:10.1371/journal.pone.0290706)
Supplement: Attachment — Submitted filename: rebuttal__26_09.pdf [file pone.0290706.s003.pdf]

Response to the reviewers

We thank the editor and the reviewers for their critical assessment of our work. In the following letter we address the concerns point by point, using *italics* for the review and **bold roman** font for our replies.

Reviewer 1

Summary
This paper tests four types of task-switching metrics to choose the optimal curriculum for continual learning. These metrics are namely, Performance, Competence, Prediction gain, and Information gain. The authors experiment with these metrics in a two-task scenario. The first task consists of standard MNIST digit classification. Then, the network presented a second task which requires summing two digits and deciding whether the sum is odd or even. The authors conclude that none of these metrics provided a signal that could guide active learning when training a network in a continual manner.

I think the overall paper is well-written and easy to follow. Furthermore, work is well-motivated based on the literature on human learning. However, I have some concerns regarding the choice of tasks. Also, I have concerns about the choice of network architecture and some of the experimental results. Please see below.

Thank you for your enthusiasm.

Comments
I suspect that Task 2 does not necessarily require higher-level abstract information compared to Task1. Suppose our network can map 1,3,5,7,9 to binary value "1" and 0,2,4,6,8 to "0". Then Exclusive Or (XOR) operation on these two binary values will give us the desired output for Task 2. Therefore, Task 2 boils down to binary classification and learning the XOR function. So I can't entirely agree with "Task 2 required the network to recognize two digits simultaneously and analyse higher-level abstract information."

Thank you for this observation. We agree that if a network could map input image to digit classes, then learning the XOR mapping would be trivial. However, mapping the images to the digit classes is complex. In Task 2a, each digit is distributed equally frequently across the odd and even (digit-sum) classes, and thus these classes (and hence the error signal) are orthogonal to the presence of any given digit. During learning, weight changes after each batch may cancel each other. This could (and empirically, does) make it impossible for the network to learn the image-to-digit mappings during Task 2a. Learning the digit representations beforehand eliminates this problem.

It is worth noting that simply splitting the curriculum into two tasks without learning them in the right order doesn't solve the problem. Specifically, learning the XOR task first is not beneficial: performance was still no better than random (see Fig.5).

We believe this situation may be often encountered, in tasks that require understanding of higher-level abstract information (such as learning language or mathematical equations) or where subgoals seemingly move the state further from the final goal (the Tower of Hanoi game). However, not all abstract tasks will require this approach and so using this term creates confusion. Therefore, the study incentive was rephrased for clarity and a new task added to further validate our theory. The "Data and tasks" section now begins:

> "Our goal was to design a task set that would benefit from training with a curriculum, but that was sufficiently simple to allow exploration of final performance across many training scenarios. The curriculum of our network consisted of two tasks. Task 1 was a simple supporting task of recognizing single-digit numbers. There was then one of two more complex main tasks. Task 2a required the network to discriminate whether the sum of two digits in the picture was odd or even. Task 2b (discussed further in section 2.3.2) required the network to calculate the sum of two digits in the picture.
>
> Task 2a could be conceptualised as two tasks that we would expect the network to be able to learn in isolation: an odd/even classification for each of the digits (which should be of a similar difficulty to digit recognition and would require the network to create single digit representations); and XOR to resolve the digit sum - *even* if both single digits are either odd or even, and *odd* if one is odd and the other even. While the network should be able to learn each of these tasks in isolation, when they are presented together, we predicted the network would fail to learn. Each digit is equally distributed among the odd-even digit-sum classes and so the error signal would be orthogonal to the presence of any given digit. We therefore predicted the network would be unable to learn the single digits (or the initial odd-even classification). Put another way, during training the weight changes each batch will cancel each other out (assuming the XOR transformation is not yet present at the higher level), which would make it difficult or potentially even impossible to learn the image-to-single digit mapping required for good task performance. We therefore predicted that pre-training on Task 1 would be a valuable stage towards optimizing performance on Task 2a, as learning the digit representations beforehand eliminates this problem.
>
> More generally, we hypothesized that such hierarchical learning scenarios will be encountered when dealing with tasks that require higher-level abstract information (such as learning language or mathematical equations) or where sub-goals seemingly require moving further away from the final goal (the Tower of Hanoi game). However, some abstract tasks may not have this property. To investigate this, we explored a variant, Task 2b, where the network had to learn the sum of digits in the picture instead of whether that sum was odd or even. Our prediction was that, although this is also a multi-digit task building upon single-digit representations with an even greater number of potential answers, it would be more easily learnable as the error signal for the multi-digit task (when classes were coded appropriately, see section 2.3.2) was not orthogonal to the single-digit classes. We hypothesized that the Task 2b error signal will thus be able guide

effective learning of the single digits."

Experiments suggest that the network cannot learn Task 2 alone. The final accuracy is 50% for binary classification, which is random. Authors suggest that this shows that this task structure was effective in requiring a curriculum. However, I firmly believe the network should be able to at least perform better than random without learning to classify digits. What is the authors' opinion regarding this issue? Based on my above comment, I strongly think the network does not necessarily need to know how to classify ten digits to perform Task 2.

The reviewer was indeed correct, that with sufficient additional training - doubling the number of epochs - it was possible to make Task 2 somewhat learnable. However, even with this doubling of computation, performance was well below that achieved with the addition of pre-training on Task 1 (see Supplementary Fig.1). More complex tasks might require an even greater multiple of increase in computation.

The overarching goal of this paper was to compare different training regimes. To provide sensitivity to the manipulations, it was important that performance was not at ceiling, and so the network architecture and data quantity were chosen to yield an imperfect level of performance. With the original number of epochs, performance on Task 2 without pre-training was on average random (Fig.1 in Supporting information). Therefore, we achieved the core design goal, to create a paradigm in which pre-training is valuable, that could then be used to evaluate training regimes, and in particular automatic curriculum selection.

Based on figure-1, convolutional layers have only one filter. What is the rationale behind this choice since it is uncommon to have only one filter in convolutional layers?

Thank you for this correction, this is a mistake in the diagram. The actual number was 64 filters. We have fixed this in a revision of Fig. 1.

Figure 4 shows that the network reaches accuracy of 80% on Task 1 (MNIST classification). This is too low for the task. Simple two-layer MLP can reach accuracy values of around 97.5%. So I think the network may not have enough capacity to learn tasks due to one filter per layer.

As noted above, to maintain sensitivity to the manipulations we designed the network so that performance was not at ceiling.

MINOR COMMENTS

(1) A figure can support the explanation of the training procedure (section 0.3).

After careful consideration we refrained from adding a figure and instead explain the training procedure in more detail instead (please see section 2.3.1 on p.5).

(2) In line 148, "n" is used for the first time, but the meaning of "n" is not explained.

Fixed, please see p.5.

(3) Numbering of sections is a little confusing. Specifically, all subsections have numbers like 0.X, which leaves an impression that all subsections belong to section 0.

Fixed.

(4) Some points in the Training procedure subsection are confusing to me. I think the main reason is the use of the word "batch". Specifically, I first thought that word "batch" referred to mini-batched used in stochastic gradient descent. However, later I interpreted word batch as mini-datasets that you used. So, I think it would help the reader if the meaning of batch is explicitly mentioned.

This was indeed referring to the mini-datasets. The phrasing was fixed for more clarity, mini-datasets are now referred to as "subsets".

(5) I think there is a typo in line 172. zaremba should be (Zaremba, 2014)

Fixed.

(6) Line 266 "Fig." is repeated twice.

Fixed.

Reviewer 2

Summary
This study presents an interesting set of experiments that focus on detecting when a sufficient amount of learning has been done on one task to benefit another future task. The authors look at simple scenario of two tasks designed using the MNIST dataset. The primary experiments explore the impact of the training on the first task (task 1) before the second more complex task (task 2).

Despite this, I felt that some of the explanation of the experimental setup could have been more clear. The exact training scenario and how the data is presented to the models in a continual versus static way is not clear. The choice of hyper-parameters also do not seem ideal for this dataset and task

and the authors provide no justification or experiments where these values are varied. This makes it difficult to comment further on the experimental results and discussion. My overall recommendation would be the following:

- make the training and evaluation protocol much more clear so that the results can be properly interpreted.

We have made the description of the training and evaluation protocol much shorter and we hope clearer.

- ensure proper use of terminology, the use of the terms "active learning" and "continual learning" appear to be used quite loosely where I think (at least as it is currently described) the work is more similar to transfer learning.

The use of terminology has been clarified and a better explanation of continual paradigm has been given. Our goal was to evaluate metrics that could drive automatic creation of curriculum, and while not being active learning in the true sense, our model encounters the same type of challenges that a model following an active learning paradigm would.

- ensure that the network architectures, training times, and hyper-parameters are appropriate for the tasks to avoid drawing incorrect conclusions.

We have extended our hyper-parameter search to include the number of epochs, in addition to the existing manipulations of learning rate and the number of convolutional layers.

Individual Points:

The authors claim in section 0.1 that the goal was to design a sufficiently simple task such that they could exhaustively explore final performance across hundreds of different training scenarios, however the experiments seem fairly limited in terms of different scenarios outside of varying the amount of iterations over the data in the two tasks.

We agree this language was misleading and we have now corrected it. "but that was sufficiently simple to allow exhaustive exploration of final performance across hundreds of different training scenarios." has been changed to "but that was sufficiently simple to allow exploration of final performance across many training scenarios"

The goal of this experiment was to investigate whether for a certain class of tasks a network would be capable of finding an optimal curriculum, given only the information available during a single training run. Exhaustive evaluation of different learning rates and architectures was not required for this.

In section 0.3, the authors discuss both continual and static training in the context of two tasks, but I was not able to find a description of the exact distinction between the two training scenarios. Are the "sequential batches of 1250 examples" distinct in terms of the classes present or are they just subsets of the entire dataset randomly chosen. Is the continual part a distinction about freezing the weights for the task 1 part of the network? Understanding exactly how the networks are trained is crucial to evaluating the results.

The description in section 3 has been rewritten, and a diagram of the training procedure has been added.

The weights are not frozen at any point of training, which has now been clarified on page 5. The distinction is between classic pre-training for the static scenario (processing all the available data of n*1250, with image class randomised from one training sample to the next) and a more naturalistic scenario for continual learning where data is only available in small sequential slices.

In section 0.6 the authors claim that Task 2 was unlearnable without some initial training on task 1 and their result seem to indicate this. However, I am curious if this may be due to the training protocol or network architecture. Did the authors varying the length of training on either tasks (number of epochs) or vary the hyper-parameters? The addition of one conv layer in the task 1 portion of the network as mentioned in section 0.2 may not be enough, but I believe that task 2 should certainly be learnable with the right scenario without any pre-training.

The reviewer was indeed correct, that with sufficient additional training - doubling the number of epochs - it was possible to make Task 2 somewhat learnable. However, even with this doubling of computation, performance was well below that achieved with the addition of pre-training on Task 1 (see Supplementary Fig.2). More complex tasks might require an even greater multiple of increase in computation.

The overarching goal of this paper was to compare different training regimes. To provide sensitivity to the manipulations, it was important that performance is not at ceiling, and so the network architecture and data quantiy were intentionally chosen to yield an imperfect level of performance. With the original number of epochs performance on Task 2, and without pre-training, performance was on average random (Fig.2 in Supporting information). Therefore, we achieved the core design goal, to create a paradigm in which pre-training is valuable, that could then be used to evaluate training regimes, and in particular automatic curriculum selection.

Minor comments

Section 0.2, line 130 - "To investigate the generalisation of switching metric performance across networks, we also tested —them— a network variant that was similar, except that it had three rather than two convolutional layers." The word them should be removed.

Fixed.

Reviewer 3

Summary
The work aims at understanding the effect of curriculum learning with fundamental tasks are followed by tasks of higher complexity, in the context of continual learning or learning down stream tasks with higher level of complexity. The authors create two tasks, wherein the first task is a simpler task and can be used to solve the more complex task 2. The authors also experiment with 4 different metrics for deciding the optimal stage to switch between tasks ,that is how much pre-training on task-1 can give you the best performance on task-2. The authors conclude that an optimal amount of pre-training on task 1 improves the performance on task two, but over-training on task-1 leads to degradation in accuracy for task-2. Further, none of the metrics experimented with to decide optimal amount of pre-training turned out to provide valuable insight.

Thank you for the excellent suggestions for task modifications and additional literature.

1. I find this work really thought provoking, however the authors miss a lot more additional motivating factors. For example, curriculum learning or task evolution has been shown to promote modularity where the ANNs learn to solve tasks with increasing complexity.

Thank you for these additions. The "Introduction" section has been expanded, please see p.2:

> **"When training a network to perform multiple tasks a correctly chosen curriculum has been shown to push the network towards either more specialized or more flexible representations without having to modify the architecture [?], affecting the emergence of alternative neural mechanisms and allowing the researchers to choose between higher performance for learned tasks at the costs of flexibility or a better performance over a wide range of tasks at the expense of a lower performance for the learned tasks.**
>
> **Decomposing a complex task into a sequence of source tasks with gradually increasing complexity has been shown to greatly improve performance for certain types of reinforcement learning [?].**
>
> **Curriculum learning has also been proven especially useful when dealing with continual learning. Modern machine learning excels at training powerful models from fixed datasets and stationary environments but these models often fail to emulate the robustness and efficiency of human learning in a non-stationary world [?, ?]. One of the most known cases is catastrophic forgetting — the rapid performance degradation on earlier learned tasks that occurs when dealing with**

highly non-stationary data — but ANN models more broadly under-perform when presented with changing or incremental data regimes [?].”

2. I find the setting of the experiment very interesting but highly unclear. I would suggest to the authors to make the setting more clear.

For example, in the continual learning setting the authors write that the ANN observes a batch of size 1250 for 3 epochs and then does not observe those samples in further training. However, does that batch contain samples from all classes ?

We have clarified this, by changing

> **“For continual learning the network learned Task 1 incrementally in n batches of 1250 examples (training on 3 epochs for each batch), which was then followed by similar continual training on n*1250 examples of Task 2. For Task 2, inside the batch the classes were balanced (each of the 45 classes was equally represented taking at least *2%* of the batch), the presentation sequence was random, with no compensation for potential repetition.”**

to

> **“For continual pre-training, n non-overlapping subsets of 1250 exemplars were sampled without replacement from the pool of 30000 MNIST digits. These subsets will have on average sampled the digits and the writers in a balanced way, but differed in their specific exemplars. Three epochs of training were conducted on the first subset, then three epochs on the second, and so on, until n subsets had been used. This ”exemplar incremental“ design reflects a weaker form of non-stationarity than is often present in the environment, but it still affected network learning substantially.”**

If so then the claims about catastrophic forgetting made later in the results section may not be true. Clarity in the setting of experiment is highly lacking.

3. An experiment to show catastrophic forgetting by providing confusion matrices on both tasks and analyzing them would be beneficial. As the setting isn’t clear this experiment may or may not be useful.

We agree, this was indeed an incorrect usage of the term catastrophic forgetting. In this paper we present a more general case of performance decline during continual learning. Due to the limitations of architecture imposed by the original study goal, ours isn’t a suitable model to deal with true non-stationarity and catastrophic forgetting. The results of training in a category-incremental way can be seen in Fig.2 of the Supporting information section. Investigating the interplay between curriculum and active learning will be the direction of our future work.

4. I would also advise adding more tasks to the pipeline, for example the first task could be classification, then addition and then a task predicting addition modulo 2. This would help in understanding what is happening wen we try to scale curriculum learning. Further, I would also suggest very simple set of tasks, which can be easily proven to be hierarchical to foster more understanding than the t-SNE plots which vary highly with variables used to create them.

Thank you for this valuable suggestion. We were particularly intriguied by the inclusion of an addition task, as this has much in common with the odd/even task (two digits presented, single digits valuable to task, abstract classes) but could be designed so that the classes in Task 2 were not orthogonal to the single digits. Our prediction, therefore, was that it would not require pre-training.

To ensure that the labels were not orthogonal to the component digits, we provided the network with a "magnitude style" encoding:

$$l_i = \begin{cases} 1, & \textbf{if } i \leq X \\ 0, & \textbf{if } i > X \end{cases}$$

where l_i is the output of neuron i, X is the sum of the two digits.

The new task is described as Task 2b. The results are shown in Fig.11 and described in the text in section 3.3: "Using digit addition for Task 2".

5. I am not convinced that the task and the architecture used are entirely correct. Is it so that when converting a single digit image to 84x84 the image is padded rather than resized? If not, then is it so that augmentations are performed while training to give scale invariance to the ANN ? If both of those are not done then the 3x3 filters re not observing the same scale of inputs when moving from task 1 to task 2. This may hinder in the ability of ANNs to learn task 2 significantly.

The image was padded and therefore no scale invariance training was needed. We have now noted this in the text to avoid possible misunderstanding (see p.4): "For the "simple" Task 1, digits were scattered on a white background 84x84 squares to ensure learning spatially invariant representations. Note the images were padded and not resized, and so scale invariant training was not needed)."

Minor comment : The figures need to be captioned, labelled and of higher resolution.

Fixed.
