# Supplementary material for: The limitations of automatically generated curricula for continual learning
Source: PLoS One. 2024 Apr 16;19(4):e0290706. doi: 10.1371/journal.pone.0290706 (PMC11020929; doi:10.1371/journal.pone.0290706)
Supplement: Attachment — Submitted filename: rebuttal_2nd_revision.pdf [file pone.0290706.s004.pdf]

Response to the reviewers

We thank the editor and the reviewers for their critical assessment of our work. In the following letter we address the concerns point by point, using *italics* for the review and **bold roman** font for our replies.

Reviewer 1

Summary

This paper tests four types of task-switching metrics to choose the optimal curriculum for continual learning. These metrics are namely, Performance, Competence, Prediction gain, and Information gain. The authors experiment with these metrics in a two-task scenario. The first task (pre-training) consists of standard MNIST digit classification. Then, the network is presented with a second task which requires summing two digits and deciding whether the sum is odd or even. The authors conclude that none of these four metrics provided a signal that could guide the learner to decide on optimal switching points.

I think the overall paper is well-written and easy to follow. Furthermore, work is thought-provoking and well-motivated. However, I have some concerns about claims, and a few details of the experiment setting are unclear to me.

Thank you for the constructive feedback on our paper and for recognizing its potential!

General

In my opinion, the given evaluation scenario is closer to transfer learning than continual learning. Continual learning comes with several desiderata, such as avoiding forgetting, learning long sequences of tasks, and forward knowledge transfer. This work only focuses on the "forward knowledge transfer" aspect of curricula for continual learning.

It does not investigate forgetting, which is one of the most important problems in continual learning. For example, what if the metric "X" does not help predict the optimized switching moment in terms of accuracy on Task 2 but provides a switching moment that leads to less forgetting?

I see that this is briefly mentioned in Supporting information and Discussion. I would suggest clarifying this distinction in the introduction by highlighting that this work only focuses on one aspect of continual learning.

Thank you for this correction.

The scope of the experiment has been further clarified in the introduction section. Additionally, as this study was inspired by an attempt to solve a problem highly specific for developmental robotics, we also further elaborated the context.

Please see page 3 (lines 87-93):

> **"This is a particular concern in developmental robotics, a scientific field which, among other things, aims at studying the developmental mechanisms and patterns by building robots able to learn from their surroundings and choose their own training input. Curiosity-driven algorithms have been shown to encounter pathological cases, such as becoming fixated on learning from completely unpredictable stochastic data (Burda et al., 2018) and in developmental robotics they can fail when the need to switch between a variety of tasks arises (Oudeyer et al., 2009). Choosing a stream of input relying on curiosity alone doesn't seem to be a viable strategy."**

And pages 3-4 (lines 114-120):

> **"The cumulative evidence thus indicates that we need a better understanding under which exact conditions automatic curriculum building/curiosity driven learning is viable. The goal of this paper is to investigate such conditions, as well as the optimal metrics to guide learning.**
>
> **In continual learning in ANNs, an assortment of goals needs to be achieved, including avoiding forgetting, learning sequences of tasks, and forward knowledge transfer. In this paper we address the problem of knowledge transfer and the problem of switching between tasks (i.e. assessing when a supporting task has been sufficiently 'learned')."**

Data and tasks

My primary concern was that Task 2 does not necessarily require higher-level abstract information compared to Task1. The Authors partially address this concern with the "Orthogonality hypothesis" and a few empirical results. Also, I understand the motivation of keeping performance below the maximum possible accuracy by restraining computation resources (e.g., the number of epochs).

However, I think the claim "potentially even impossible to learn" (line 134) is inaccurate and needs to be revised. Suboptimal accuracy or requirement for more resources differs from failing to learn. Furthermore, in "S1 Fig.1" the authors show that Task 2 is learnable (but with worse accuracy) if the number of epochs is changed. I strongly believe that Task 2 can even be learned with fever epochs with higher accuracy with a more optimal training routine (e.g., learning rate schedule, weight decay, proper batch size).

Thank you for this observation. This is indeed the case, this particular task can be made learnable with minor tweaks to network architecture or learning regime.

However, generally speaking, there could potentially exist more complex tasks with similar properties, which, while theoretically learnable, would in practice require adjustments that would be too computationally expensive.

We have therefore removed the "potentially impossible to learn" claim and added clarification on page 4 (lines 149-153):

> **"In this simple model a similar result could potentially be achieved by optimising the amount of training epochs or modifying the architecture of the network. However, in real life applications and with more complex tasks with similar properties this could require significant computational resources to adjust the network in an effective way. As the The Nevis'22 Benchmark experiments (Bornschein et al, 2022) show, the assessment of the compute spent while learning should include the compute spent while doing hyper-parameter search to evaluate the models effectively. This makes the optimization provided by the curriculum a welcome addition."**

Model

(1) In the first sentence, the number of filters of convolutional layers is not given. I think it should be also presented here (I see it is given in Figure 1) to understand the model's learning capacity better.

The number of filters has been added to the text.

(2) I think the CNN visualization in Figure 1 is confusing. For example, the input size and the number of filters are given as dimensions of the same shape. In deep learning literature, it is more common to have LeNet or AlexNet style visualizations – please see https://alexlenail.me/NN-SVG/LeNet.html and https://alexlenail.me/NN-SVG/AlexNet.html.

Figure 1 has been adjusted, the network is now visualized in the LeNet style.

(3) The authors propose to train output layer-1 for task-1 and a second output layer that follows output layer-1 for task-2. I think a justification for this choice should be provided because it is not common to have such output structures in continual or transfer learning. Specifically, either independent task output heads are used for different tasks (e.g., continual learning multi-head scenario), or the previous task output layer is replaced with a new one (e.g., transfer learning with different targets).

As we used a network with only 2 convolutional layers, we were unsure whether proper representations could be formed before the final dense layer and whether removing them might have further hindered the network's ability to learn Task 2. In the future experiments, as we move to more naturalistic tasks and deeper networks, we plan to use a more conventional output structure.

We have therefore added clarification to the text (please see page 5, lines 191-195):

> **"Adding a second output layer that follows output layer-1 for Task 2 was also dictated by the performance not being at ceiling. As we used a network with only 2 convolutional layers, we were cautious of the possibility that proper digit**

representations might only be formed in the final dense layer and thus removing them would further hinder network's ability to learn Task 2. "

Training procedure

Some hyperparameters (e.g., optimizer, weight decay, learning rate) and how they are determined are unclear. I suggest that the authors mention such details in this subsection.

A description of hyperparameters has been added, please page 6.

Supporting information

(1) S1 Fig.1 Effect of the number of epochs. Legend is missing in the plot. Does the blue line double the number of epochs?

This is indeed so, the figure has been updated with the correct legend.

(2) S2 Fig.1 Catastrophic forgetting scenario. The sentence "Class-incremental learning during pre-training learned to a catastrophic forgetting scenario that couldn't have been solved simply with switching at the correct time." is not clear to me. I suggest that the authors rephrase this part.

The description has been rephrased to "Class-incremental learning during pre-training led to a catastrophic forgetting scenario where the network never learned single digits representation enough to facilitate learning of Task 2".

(3) Furthermore, the term "Class-incremental learning" is used for a specific continual learning scenario where the learner encounters novel classes in a sequential task and requires predicting all the previous classes regardless of task knowledge. However, if my understanding is correct, all ten digits are presented to the learner during pretraining. So, it is not a class-incremental learning scenario.

In the supporting information we are reviewing specifically the case of class-incremental learning, as opposed to the procedure described in the main body of the paper. Here Task 1 can be seen as a sequence of smaller tasks where each digit is learned after the other. The analysis in the support section was performed to explain why does catastrophic forgetting fall outside of the scope of this experiment: the restrictions placed on our network by the main research question are too strict, rendering it unable to deal with forgetting.

Reviewer 2

My major concerns from the original submission have been addressed and the work now has a clear description of the experimental protocol. The design of the experiment still seems a bit weak to me, but it does still support the conclusions found by the authors. Overall, the work is ready to be accepted.

Thank you for recommending the paper to be accepted! Your comments and suggestions have greatly improved the quality of our work and we will strive to investigate the topic more thoroughly in the future with better experiment design.

Reviewer 3

I would like to thank the authors for addressing all the previous concerns. The writing has been improved drastically and many more details have been added (which were lacking) making the manuscript more thorough.

Thank you for your comments and suggestions, your previous suggestions indeed helped to fill many gaps in our manuscript.

The only concern that remains is that of lacking evidence regarding the conclusion that amount of training on Task-1 has a drastic effect on Task-2. Although I agree with the main experiment conducted in the work, I would also like to see more evidence for the same through another experiment with different tasks but with increasing complexity. Example : MNIST digit classification can be Task-1, and the Task-2 can be class specific rotation, where depending on the class the input image is rotated by some fixed angle. Class-1 : angle 30, Class-2 : angle 45 etc.

We would like to express our gratitude for the time and effort you have dedicated to reviewing our manuscript, your previous suggestions have been extremely helpful in filling a theoretical gap in our work.

However, after careful consideration, we believe that incorporating this new experiment might fall outside the scope of the current paper. Our main objective in this study is to focus on providing an example where curriculum is beneficial for learning however cannot be found by the existing curiosity metrics.

We believe that the optimal way of generalising this result would be to create a continuum of tasks of varying complexity rather than finding another isolated task. This is a topic of our current research, however, it would require a separate study with a completely new set of experiments to properly address this problem.

We respectfully request the possibility of waiving the requirement for this revision, if feasible.
